# Supplementary figures and images for: Distribution, antifungal susceptibility pattern and intra-Candida albicans species complex prevalence of Candida africana: A systematic review and meta-analysis
Source: PLoS One. 2020 Aug 20;15(8):e0237046. doi: 10.1371/journal.pone.0237046 (PMC7440629; doi:10.1371/journal.pone.0237046)

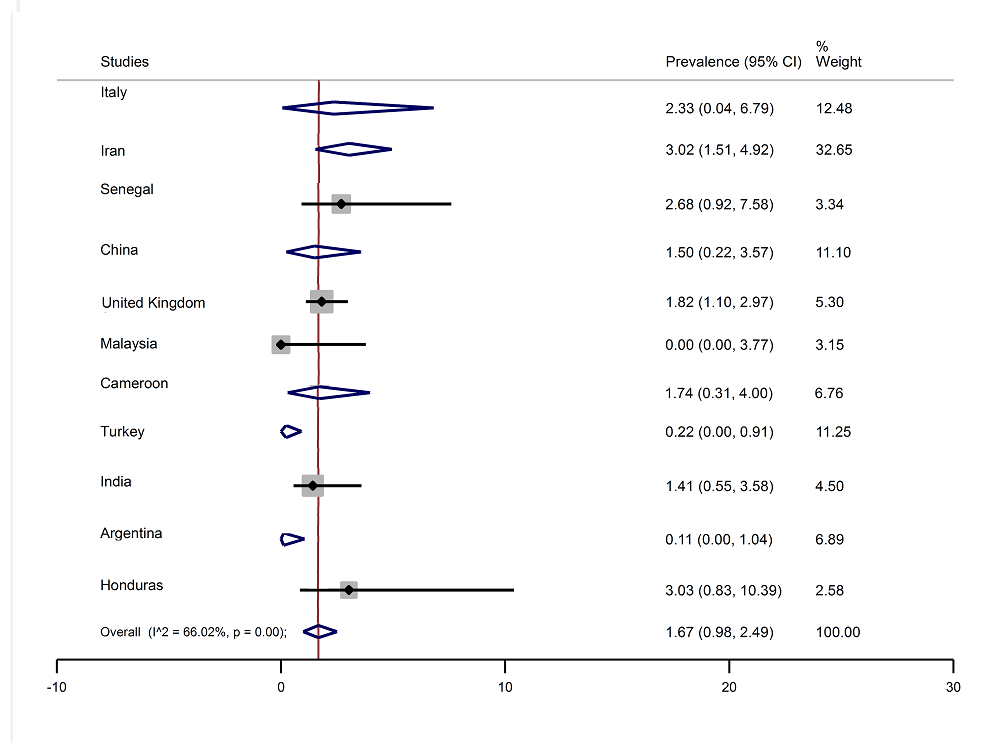

Supplement: S1 Fig — (TIF) [file pone.0237046.s001.tif]

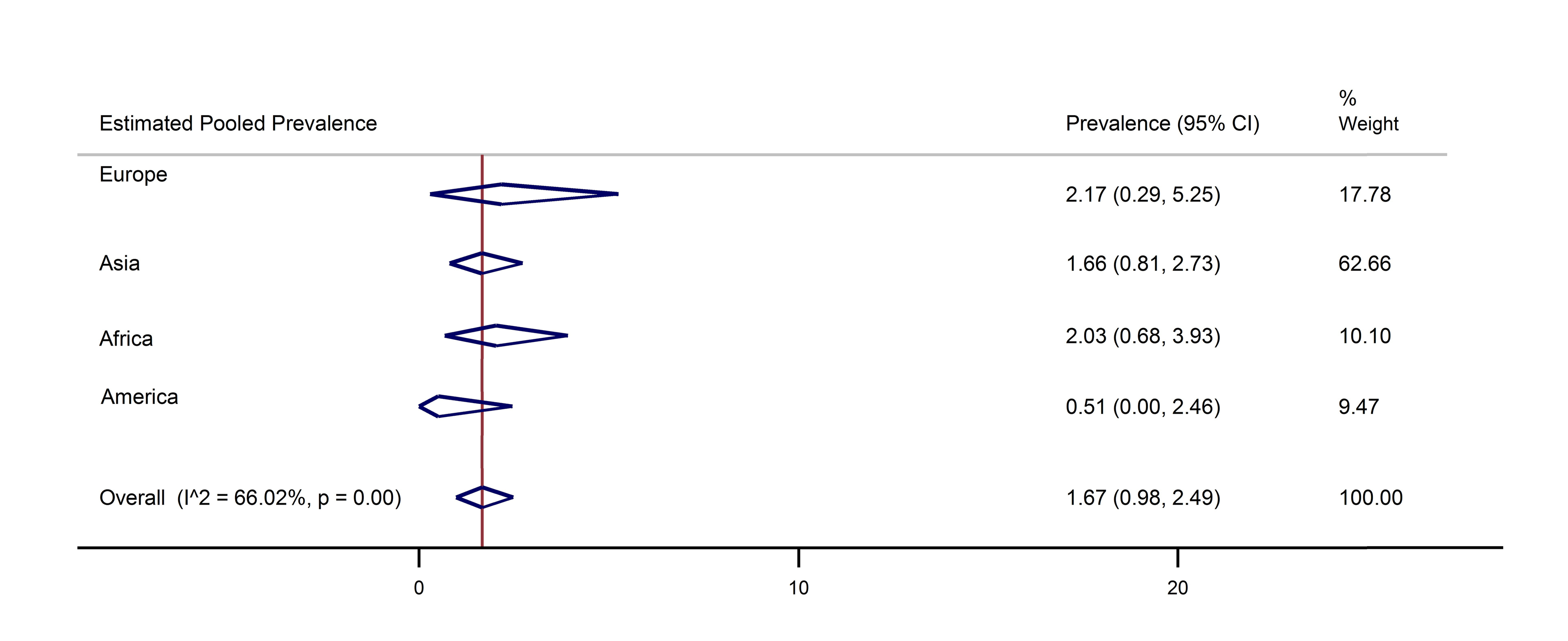

Supplement: S2 Fig — (TIF) [file pone.0237046.s002.tif]

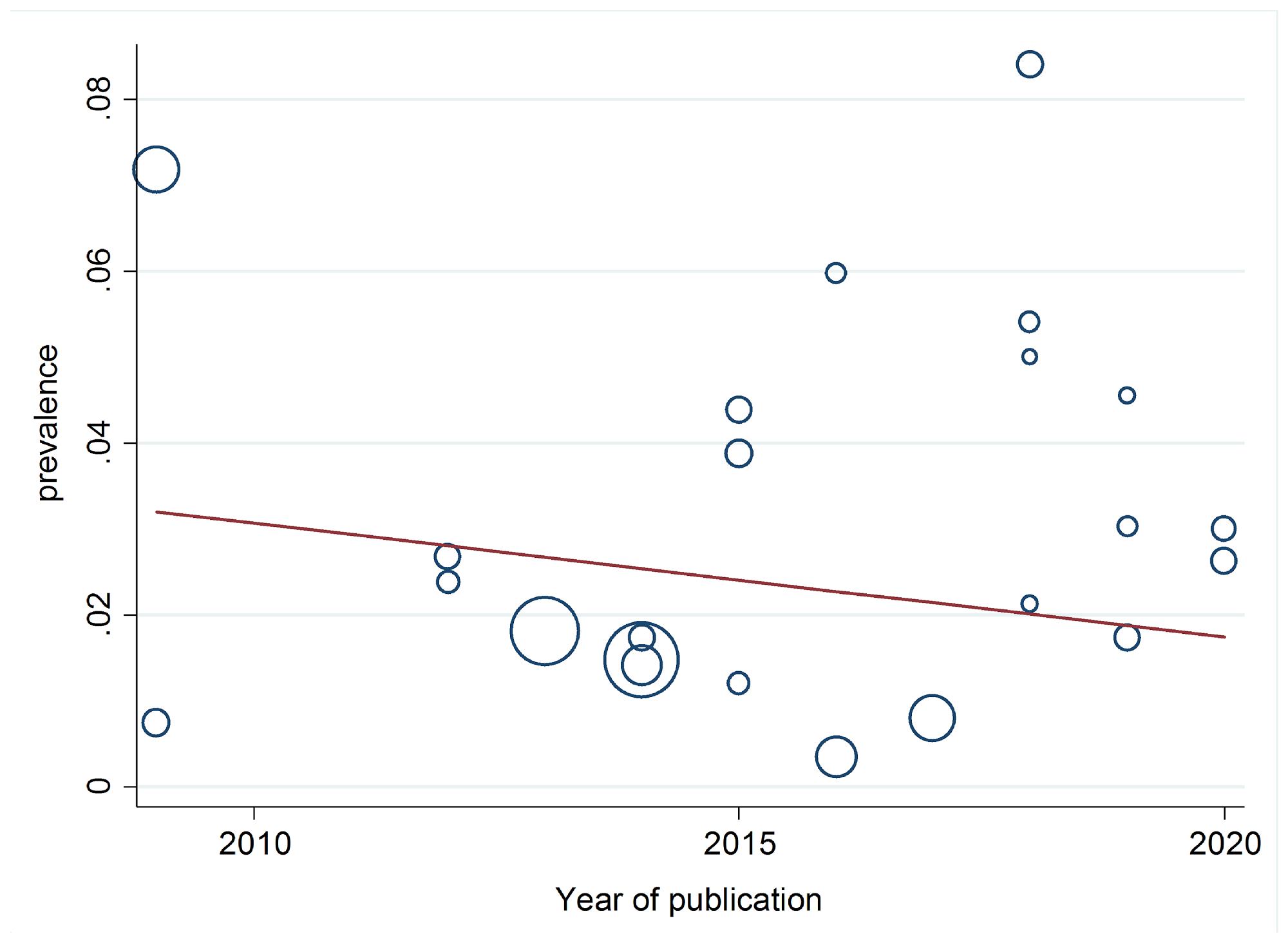

Supplement: S3 Fig — (TIF) [file pone.0237046.s003.tif]
